# The gene normalization task in BioCreative III

**DOI:** 10.1186/1471-2105-12-S8-S2

**Published:** 2011-10-03

**Authors:** Zhiyong Lu, Hung-Yu Kao, Chih-Hsuan Wei, Minlie Huang, Jingchen Liu, Cheng-Ju Kuo, Chun-Nan Hsu, Richard Tzong-Han Tsai, Hong-Jie Dai, Naoaki Okazaki, Han-Cheol Cho, Martin Gerner, Illes Solt, Shashank Agarwal, Feifan Liu, Dina Vishnyakova, Patrick Ruch, Martin Romacker, Fabio Rinaldi, Sanmitra Bhattacharya, Padmini Srinivasan, Hongfang Liu, Manabu Torii, Sergio Matos, David Campos, Karin Verspoor, Kevin M Livingston, W John Wilbur

**Affiliations:** 1 National Center for Biotechnology Information (NCBI), 8600 Rockville Pike, Bethesda, Maryland 20894, USA; 2 Department of Computer Science and Information Engineering, National Cheng Kung University, Tainan, Taiwan, R.O.C; 3 Department of Computer Science and Technology, Tsinghua University, Beijing, 100084, China; 4 Institute of Information Science, Academia Sinica, Taipei 115, Taiwan; 5 Information Science Institute, University of Southern California, Marina del Rey, California, USA; 6 Department of Computer Science and Engineering, Yuan Ze University, Chung-Li, Taiwan, R.O.C; 7 Department of Computer Science, National Tsing-Hua University, Hsinchu, Taiwan, R.O.C; 8 Institute of Information Science, Academic Sinica, Taipei, Taiwan, R.O.C; 9 Interfaculty Initiative in Information Studies, University of Tokyo, Japan; 10 Graduate School of Information Science and Technology, University of Tokyo, Japan; 11 Faculty of Life Sciences, University of Manchester, Manchester, M13 9PT, UK; 12 Department of Telecommunications and Media Informatics, Budapest University of Technology and Economics, 1117 Budapest, Hungary; 13 Medical Informatics, University of Wisconsin-Milwaukee, Milwaukee, Wisconsin, USA; 14 BiTem Group, Division of Medical Information Sciences, University of Geneva, Switzerland; 15 BiTeM Group, Information Science Department, University of Applied Science, Geneva, Switzerland; 16 NITAS/TMS, Text Mining Services, Novartis AG, Switzerland; 17 Institute of Computational Linguistics, University of Zurich, Zurich, Switzerland; 18 Department of Computer Science, The University of Iowa, Iowa City, Iowa 52242, USA; 19 Department of Health Sciences Research, Mayo Clinic College of Medicine, Rochester, MN 55905 USA; 20 Lab of Text Intelligence in Biomedicine, Georgetown University Medical Center, 4000 Reservoir Rd., NW, Washington, DC 20057 USA; 21 DETI/IEETA, University of Aveiro, Campus Universitário de Santiago, 3810-193 Aveiro, Portugal; 22 Center for Computational Pharmacology, University of Colorado School of Medicine, Aurora, Colorado, USA

## Abstract

**Background:**

We report the Gene Normalization (GN) challenge in BioCreative III where participating teams were asked to return a ranked list of identifiers of the genes detected in full-text articles. For training, 32 fully and 500 partially annotated articles were prepared. A total of 507 articles were selected as the test set. Due to the high annotation cost, it was not feasible to obtain gold-standard human annotations for all test articles. Instead, we developed an Expectation Maximization (EM) algorithm approach for choosing a small number of test articles for manual annotation that were most capable of differentiating team performance. Moreover, the same algorithm was subsequently used for inferring ground truth based solely on team submissions. We report team performance on both gold standard and inferred ground truth using a newly proposed metric called Threshold Average Precision (TAP-*k*).

**Results:**

We received a total of 37 runs from 14 different teams for the task. When evaluated using the gold-standard annotations of the 50 articles, the highest TAP-*k* scores were 0.3297 (*k*=5), 0.3538 (*k*=10), and 0.3535 (*k*=20), respectively. Higher TAP-*k* scores of 0.4916 (*k*=5, 10, 20) were observed when evaluated using the inferred ground truth over the full test set. When combining team results using machine learning, the best composite system achieved TAP-*k* scores of 0.3707 (*k*=5), 0.4311 (*k*=10), and 0.4477 (*k*=20) on the gold standard, representing improvements of 12.4%, 21.8%, and 26.6% over the best team results, respectively.

**Conclusions:**

By using full text and being species non-specific, the GN task in BioCreative III has moved closer to a real literature curation task than similar tasks in the past and presents additional challenges for the text mining community, as revealed in the overall team results. By evaluating teams using the gold standard, we show that the EM algorithm allows team submissions to be differentiated while keeping the manual annotation effort feasible. Using the inferred ground truth we show measures of comparative performance between teams. Finally, by comparing team rankings on gold standard vs. inferred ground truth, we further demonstrate that the inferred ground truth is as effective as the gold standard for detecting good team performance.

## Background

The gene normalization (GN) task in BioCreative III is similar to past GN tasks in BioCreative I and [1-3] in that the goal is to link genes or gene products mentioned in the literature to standard database identifiers. This task has been inspired in part by a pressing need to assist model organism database (MOD) literature curation efforts, which typically involve identifying and normalizing genes being studied in an article. For instance, Mouse Genome Informatics recently reported their search and evaluation of potential automatic tools for accelerating this gene finding process [4].

Specifically, this year’s GN task was to have participating systems return a list of gene database (Entrez Gene in this case) identifiers for a given article. There are two differences from the past BioCreative GN challenges:

• Instead of using abstracts, full-length articles were provided.

• Instead of being species-specific, no species information was provided.

Both changes made this year’s challenge event closer to the real literature curation task in MODs where humans are given full text articles without prior knowledge of organism information in the article.

Two additional new aspects of this year’s GN task were the proposed evaluation metrics and the use of an Expectation Maximization (EM) algorithm for choosing which test articles to be manually annotated when developing a gold standard and for inferring ground truth based on team submissions. As many more genes are found in full text than in abstracts, returning genes by predicted confidence is preferred to a random order, as the former is more desirable in real-world applications. Metrics used in past GN tasks such as Precision, Recall, and F-measure do not take ranking into consideration. Thus, we proposed to use a new measure called Threshold Average Precision (TAP-*k*), which is specifically designed for the measurement of retrieval efficacy in bioinformatics [5].

Finally, unlike in previous GN tasks where all abstracts in the test set were completely hand annotated, the cost of manual curation on full text prevented us from obtaining human annotations for all 507 articles in the test set. Thus we resorted to obtaining gold-standard human annotations on 50 selected articles in the test set that are best able to differentiate team results. We chose such a set of 50 articles by using an EM algorithm. Furthermore, the same EM algorithm was used for inferring ground truth over the entire test set of 507 articles based on team submissions in lieu of human annotations. That is, given a labelling task and *M* independent labelling sources, it is possible to use these multiple sources to make estimates of the true labels which are generally more accurate than the labels from any single source alone. Perhaps the simplest approach to this is to use majority voting[7,8]. On the other hand a number of methods have been developed using latent variables to represent in some way the quality of the labelling sources and based on the EM algorithm[10-13]. There is evidence that such an approach can perform better than majority voting [9,12]. We have chosen the most direct and transparent of the EM approaches [12] to apply to the GN task where we have multiple submissions from different teams as the multiple labelling sources. Although there was a prior effort on harmonizing annotations from different automatic systems for the goal of constructing a large-scale annotated corpus in biomedicine [14], as far as we are aware this is the first attempt to base an evaluation of the performance of multiple computer algorithms on an EM algorithm for multiple independent data sources. As shown in the Results Section, the inferred ground truth (also referred to as *silver standard* in this paper) is as effective as the gold-standard human annotations in differentiating good team performance.

## Methods

### Data preparation

For the purpose of obtaining full text articles in a uniform format and using them as a source for text analytics, all the articles selected for this task were published either by BioMed Central (BMC) or by Public Library of Science (PLoS), two PubMed Central (PMC) participating Open Access publishers. As a result, the text of each article was readily made available in both high-quality XML and PDF from PMC.

Participants were given a collection of training data to work with so that they could adjust their systems to optimal performance. The training set included two sets of annotated full-length articles:

• 32 fully annotated articles by a group of invited professional MOD curators and by a group of bioinformaticians from the National Center for Biotechnology Information (NCBI). Both groups were trained with detailed annotation guidelines (See Additional file 1) and a small number of example articles before producing gold-standard annotations. The 32 articles were manually selected to match the general species distribution in the literature as well as the domain expertise of our MOD curators. For each article in this set, a list of Entrez Gene ids was provided.

• A large number (500) of partially annotated articles. That is, not all genes that are mentioned in an article were annotated, but only the most important ones that within the scope of curation were annotated by human indexers at the National Library of Medicine (NLM). It was noted that most of the annotated genes were taken from the abstracts, though this was not 100%. This does not necessarily mean that the remainder of the text is useless. Presumably the full text can help to decide which genes are most important in the paper and determine the species to improve the prediction of the gene identifier.

For evaluating participating systems, we prepared a set of 507 articles as the test set. These articles were recently published and did not yet have any curated gene annotations. Due to the cost of manual curation, the same groups of curators were asked to produce human annotations only for a subset of 50 articles selected by the algorithm described below.

### EM algorithm

In this scheme we assume there are *M* labelling sources and associate with the *i*th labelling source two numbers, the sensitivity *as_i_* and the specificity *bs_i_*. For the GN task we consider each participating team as a labelling source and all the gene ids returned by the *M* teams as objects to be labeled. Any given team produces a label for any such gene id which is the label “true” if the team returned that gene id or “false” if the source did not return that gene id. Then the sensitivity *as_i_* is the probability that the *i*th team labels a correct gene id as true and the specificity *bs_i_* is the probability that it labels an incorrect gene id as false. Assume there are *N* gene ids which require labelling. Then the model assumes a probability distribution  where *p_j_* is the probability that the *j*th gene id is correct. To begin the algorithm we initialize each *p_j_* to be equal to the fraction of the *M* labels that are true for that gene id. The maximization step redefines the  in terms of the current  by(1)

where we have used typical Laplace smoothing and define *δ_ij_* to be 1 if the *i*th team labels the *j*th gene id as true and 0 otherwise. The *p_j_*s are defined for the subsequent expectation step by(2)

by Bayes’ theorem where for each *j*, *pr_j_* is the prior for *p_j_*. We initially took *pr_j_* uniformly to be 0.5 and applied the algorithm to choose the 50 documents for hand annotation. Once we knew the correct annotations for the 50 document gold standard set we observed that only about 1% of gene ids returned by systems were correct. We subsequently have taken *pr_j_* equal to 0.01 for all *j* in applying the algorithm to infer ground truth over all the 507 documents in the test set.

As mentioned above, our first use of this model was to find 50 documents among the 507 test documents which had the most variability in their labelling by different sources. For this purpose one submission from each team involved in the GN task was randomly selected and these submissions were the 14 sources for application of the algorithm. When the algorithm was run to convergence we computed the entropy for the *j*th gene id by the formula(3)

Each document was scored by the sum of the entropies for all the gene ids coming from that document. Thus a document score is a function of how many gene ids are reported for that document and how variably the gene ids are reported by the different sources. This sampling, running the model and scoring the documents, was repeated 100 times and the top 50 documents varied only a small amount from run to run. We chose the 50 documents with the highest average scores over the 100 trials for hand annotation to provide the *gold standard* evaluation.

The second use of the model was to apply it to the best submission from each team for inferring ground truth (silver standard) over the entire test set of 507 documents. From the converged model using the 14 sources we obtained a set of probabilities  and we accepted as correct all those gene ids for which *p_j_* ≥ 0.5 and considered all other gene ids to be incorrect. Note that the choice of the best submission itself is based on the gold standard, but this in no way makes use of relative team performance or ranking. We made no further use of the gold standard in producing the silver standard. That is, the gold and silver standards are based on completely separate sources: the human annotation and team submission, respectively. Yet, as shown in the Team Results subsection where we directly compare evaluation results using the gold standard *vs.* silver standard, they are equally effective in detecting good team performance.

### Evaluation metrics

As mentioned earlier, we use a new metric, Threshold Average Precision (TAP-*k*), for evaluating team performance. We decided to use such a metric because it can better reflect and evaluate the usability of computer-generated results in assisting human curators. Unlike F-measure—the official measure in the past two BioCreative GN tasks—it is able to consider rankings in predicted results as well as confidence scores assigned with each prediction, both of which are highly desired features for humans to make use of computer-generated predictions in realistic use cases. For instance, based on the discussion among the professional MOD curators from the BioCreative III User Advisory Group (UAG), it is unacceptable for a human curator to look through many wrong predictions to find a correct one. In other words, one is likely to lose confidence in a list of predictions if the top returned predictions are incorrect. Thus a key for computer-generated predictions to be accepted by human curators is whether or not it can help improve their productivity. Therefore, human curators can only tolerate a certain number of errors in computer-generated predictions because inspecting erroneous predictions would only slow them down otherwise. Although the level of tolerance varies between individuals, it is common for them to stop inspecting prediction results after encountering about 5 errors in most cases. Furthermore, it was also agreed by the UAG members that the confidence score associated with each prediction is useful when reviewing predictions.

For this purpose, there have been efforts to take ranking into consideration when evaluating prediction results. One suggestion was to only evaluate a fixed number of top returned results using F-measure. However, in reality it is difficult to decide the cut-off position because different documents can have widely differing numbers of genes. Another approach was used in BioCreative II.5 where the organizers adopted the area under the interpolated precision / recall curve (AUC iP/R). However this measure rewards recall because even low-ranked results can contribute to the overall AUC iP/R score. Therefore, teams were incentivized to provide as many right answers as possible even if they were low ranked, which is generally not acceptable for human use. Our response to these problems has been to propose the Threshold Average Precision (TAP-*k*) metric, as developed by Carroll et al. [5]. TAP-*k* is a derivative of Mean Average Precision—a commonly used metric for evaluating retrieval efficacy in the field of information—with a threshold determined by the first *k* errors in a ranked list. TAP-*k* is able to measure ranking, reflect the user tolerance of prediction errors (false positives), as well as make use of confidence scores. Below we show how TAP-k is computed.

For a single query the average precision (*AP*) is computed by summing the precision at each rank that contains a true positive item and then dividing this sum by the number of positives (*P*) for that query. If the retrieval system assigns to each retrieved item a score and the retrieved items are ranked in decreasing order of score, then it may be useful to cut off the retrieval at some fixed score level *x*. We can compute the average precision with cutoff *x* (*APC_x_*). This is the sum of the precision at each rank with a true positive item and a score >=*x*, divided by the total number of positives for the query. Finally, suppose that *y*>*x* and further suppose there are no true positive items in the sum for *APC_x_* that are below *y*. Then *APC_y_*=*APC_x_*. But clearly it would make more sense to choose the cutoff *y* than the cutoff *x*. To distinguish between these two cases one defines the average precision with cutoff *x* and terminal penalty (*APCP_x_*). Let *P_x_* be the precision at the last rank with score >= *x* and let *P* be the total number of positives. Then define(4)

*APCP_x_* is just the weighted average of *APC_x_* and *P_x_* with most of the weight applied to *APC_x_*, but *P_x_* supplying the terminal penalty. In our hypothetical case *P_y_* will be greater than *P_x_* so that *APCP_y_* is also greater than *APCP_x_* and the score rewards the better choice of cutoff or equally, penalizes the poorer choice. Whereas *MAP* is the average of *AP* over all the queries, *TAP-k* is the average of *APCP_x_* over all the queries where *x* is chosen as the largest score that produces a median of *k* false positive retrievals over all the queries. The median is used here instead of the mean because it is more robust against noise and outliers.

We refer interested readers to see Additional file 2 and the original publication [5] for more detailed description and some examples of the TAP-*k* metric. In the evaluation of the GN task, we used three values of *k*: 5, 10 and 20 to reflect different human tolerance of prediction errors.

## Results

### GN annotation data

As shown in Table 1, the mean and median numbers of annotated genes per article in Set 1 are significantly lower than those in Set 2, while remaining relatively close to their counterparts in Set 3. This comparison suggests that the 50 selected articles are not representative of the articles in the training set. Instead, the entire test set seems akin to the 32-article training set in this respect.

**Table 1:** Statistics of annotated gene ids in the different data sets.

Set	Description	Min	Max	Mean	Median	St.dev.
1	Training Set (32 articles)	4	147	19	14	24
2	Test Set (50 articles – gold standard)	0	375	33	19	63
3	Test Set (507 articles – silver standard)	0	375	18	12	27

Table 2 shows that there are many different species involved in this year’s GN task, which suggests that species identification and disambiguation may be critical in the process of finding the correct gene ids. We also show that the distributions of species among the genes in the three data sets are not the same. This indeed reflects the method of selecting the articles for training and evaluation: with some prior knowledge of a papers’ species information, we were able to select the 32 articles as the training set to match the domain expertise of those invited professional MOD curators in order to obtain best possible human annotations. On the other hand, the articles in the test set were selected without regard to species as none was annotated prior to the evaluation. In addition to recognizing various species in free text, participating systems also needed to properly link them to the corresponding gene mentions in the articles. As shown in Figure 1 most articles (over 70%) in our data sets contain more than one species mention. In fact, it is not uncommon to see 5 or more species in an article. In cases where more than one species is found in an article, it can be challenging for systems to associate a gene mention with its correct species.

**Table 2:** Statistics of species distribution in the different data sets.

#	Training Set (32 articles)	Test Set (50 articles)	Test Set (507 articles)
1	S. cereviaiae (27%)	Enterobacter sp. 638 (23%)	H. Sapiens (42%)
2	H. sapiens (20%)	M. musculus (14%)	M. musulus (24%)
3	M. musculus (12%)	H. Sapiens (11%)	D. melanogaster (6%)
4	D. melanogaster (10%)	S. pneumoniae TIGR4 (9%)	S. cerevisiae S228c (6%)
5	D. rerio (7%)	S. scrofa (5%)	Enterobacter sp. 638 (4%)
6	A. thaliana (5%)	M. oryzae 70-15 (4%)	R. norvegicus (4%)
7	C. elegans (3%)	D. melanogaster (4%)	A. thaliana (2%)
8	X. laevis (3%)	R. norvegicus (3%)	C. elegans (2%)
9	R. norvegicus (2%)	S. cerevisiae S228c(2%)	S. pneumoniae TIGR4 (2%)
10	G. gallus (2%)	E. histolytica HM-1 (2%)	S. scrofa (1%)
11+	Other 18 species (9%)	Other 65 species (23%)	Other 91 species (7%)

**Figure 1:**
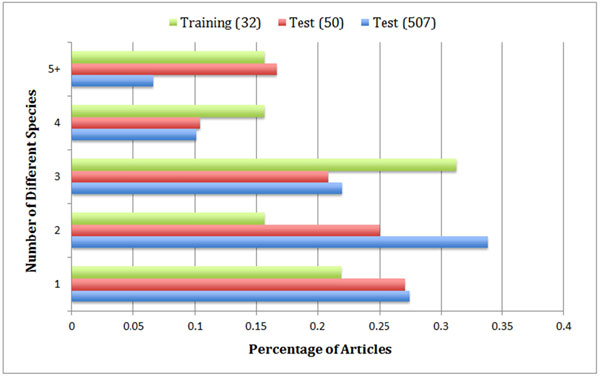
Percentage of articles annotated with different numbers of species in various data sets. Training (32) refers to the human annotations on the 32 articles in the training set. Test (50) and Test (507) refer to the gold standard and silver standard annotations on the 50 and 507 articles in the test set, respectively.

### Team results

Each team was allowed to submit up to 3 runs. Overall, we received a total of 37 runs from 14 teams. One team withdrew their late submission (one run) before the results were returned to the teams. Thus, per their request we do not report their system performance in the tables below. Nevertheless we included their withdrawn run when selecting 50 articles and computing the silver standard by our EM algorithm, as we believe more team submission data are preferable in this case.

We assessed each submitted run by comparing it against the gold standard, and report their corresponding TAP scores (*k* = 5, 10, and 20) in Table 3. As highlighted in the table, the two runs from team 83 (T83_R1 and T83_R3) achieved highest TAP scores when evaluated on the gold standard. The second best results were submitted by Team 98 (T98_R3). However, we did not find a statistically significant difference between the results of the two teams (T83 and T98) when comparing their respective best runs (with different values of *k*) based on the Wilcoxon signed rank test.

**Table 3:** Team evaluation results on the gold standard annotations of 50 documents. Results are sorted by team numbers. All gold standard annotations were provided directly by humans.

Team_Run	TAP (K=5)	TAP (K=10)	TAP (K=20)
T63_R1	0.0340	0.0488	0.0725
T63_R2	0.0296	0.0458	0.0643
T65_R1	0.0714	0.0986	0.1048
T65_R2	0.0915	0.1097	0.1183
T68_R1	0.1621	0.1876	0.2049
T68_R2	0.1285	0.1460	0.1782
T70_R1	0.0566	0.0566	0.0566
T70_R2	0.0622	0.0622	0.0622
T70_R3	0.0718	0.0718	0.0718
T74_R1	0.2137	0.2509	0.2509
T74_R2	0.2083	0.2480	0.2480
T74_R3	0.2099	0.2495	0.2495
T78_R1	0.0584	0.0741	0.1129
T78_R2	0.0847	0.1202	0.1706
T78_R3	0.0847	0.1128	0.1426
T80_R1	0.1084	0.1581	0.1646
T80_R2	0.0382	0.0516	0.0588
T80_R3	0.0329	0.0437	0.0521
**T83_R1**	0.3254	**0.3538**	**0.3535**
T83_R2	0.3216	0.3435	0.3435
**T83_R3**	**0.3297**	0.3514	0.3514
T89_R1	0.1205	0.1205	0.1363
T89_R2	0.1553	0.1553	0.1652
T89_R3	0.1295	0.1548	0.1548
T93_R1	0.1651	0.1902	0.2075
T93_R2	0.1560	0.1858	0.2062
T93_R3	0.1662	0.1916	0.2096
T97_R1	0.0727	0.0939	0.1026
T97_R2	0.0649	0.0872	0.0974
T97_R3	0.0727	0.0939	0.1026
T98_R1	0.2835	0.3012	0.3103
T98_R2	0.2909	0.3079	0.3087
T98_R3	0.3013	0.3183	0.3303
T101_R1	0.1896	0.2288	0.2385
T101_R2	0.1672	0.2150	0.2418
T101_R3	0.1812	0.2141	0.2425

To assess the quality of the silver standard, we first show in Table 4 the results of team submissions against the silver standard on the same set of 50 selected articles in order to provide a direct comparison. It is important to note that although the same 50 articles were used again in Table 4, their annotations are completely independent of the human annotations (i.e. gold standard) used in Table 3. Instead, they are simply part of the silver standard annotations obtained through the EM algorithm (see details in the Method Section). In addition, we also show in Table 4 the evaluation results of using the silver standard over all 507 articles. We expect to see some difference in TAP-*k* results when using silver standard with different numbers of test articles. Although the two best runs from Team 83 in Table 3 are still among the ones with the highest TAP scores, they no longer are the best runs. Instead, the top positions are replaced by T74_R3 and T98_R3, respectively.

**Table 4:** Team evaluation results on the 50 and 507 articles using the silver standard annotations. Results are sorted by team numbers. All silver standard annotations were derived by the EM algorithm applied to team submissions over the full set of 507 test articles. The silver-standard annotations of the 50 selected articles (columns 2-4) are taken from the silver-standard annotations obtained on the 507 articles.

Team_Runs	Using silver standard (50 selected articles)			Using silver standard (All 507 articles)		
	
	TAP (K=5)	TAP K=10	TAP (K=20)	TAP (K = 5)	TAP (K = 10)	TAP (K = 20)
T63_R1	0.0504	0.1059	0.1438	0.1584	0.1961	0.1980
T63_R2	0.0393	0.0998	0.1355	0.1415	0.1890	0.1982
T65_R1	0.1039	0.1302	0.1532	0.1549	0.1818	0.2030
T65_R2	0.1133	0.1360	0.1581	0.1573	0.1868	0.2097
T68_R1	0.2282	0.2768	0.3221	0.3614	0.3787	0.3753
T68_R2	0.2136	0.2978	0.2978	0.3468	0.3641	0.3608
T70_R1	0.0130	0.0130	0.0130	0.1227	0.1227	0.1227
T70_R2	0.0166	0.0166	0.0166	0.1323	0.1323	0.1323
T70_R3	0.0560	0.0560	0.0560	0.1579	0.1579	0.1579
T74_R1	0.3820	0.3820	0.3820	0.4873	0.4873	0.4873
T74_R2	0.3855	0.3855	0.3855	0.4871	0.4871	0.4871
**T74_R3**	**0.3890**	0.3890	0.3890	**0.4916**	**0.4916**	**0.4916**
T78_R1	0.0552	0.0786	0.1152	0.1237	0.1529	0.1900
T78_R2	0.1058	0.1592	0.2166	0.2561	0.2751	0.2751
T78_R3	0.0979	0.1440	0.1997	0.2273	0.2765	0.2872
T80_R1	0.2579	0.2840	0.2840	0.4056	0.4056	0.4056
T80_R2	0.0716	0.1150	0.1220	0.2281	0.2281	0.2281
T80_R3	0.0792	0.1269	0.1329	0.2332	0.2397	0.2397
T83_R1	0.3567	0.3600	0.3600	0.4591	0.4591	0.4591
T83_R2	0.3291	0.3291	0.3291	0.4323	0.4323	0.4323
T83_R3	0.3382	0.3382	0.3382	0.4327	0.4327	0.4327
T89_R1	0.1767	0.2251	0.2251	0.2783	0.3111	0.3111
T89_R2	0.2161	0.2617	0.2992	0.2721	0.3057	0.3057
T89_R3	0.2091	0.2091	0.2091	0.2977	0.2977	0.2977
T93_R1	0.2614	0.3093	0.3093	0.4039	0.4039	0.4039
T93_R2	0.2101	0.2625	0.2966	0.3709	0.3820	0.3820
T93_R3	0.2553	0.3048	0.3048	0.4061	0.4061	0.4061
T97_R1	0.1094	0.1317	0.1566	0.1396	0.1676	0.1918
T97_R2	0.0858	0.1133	0.1352	0.1344	0.1601	0.1829
T97_R3	0.1094	0.1317	0.1566	0.1396	0.1676	0.1918
T98_R1	0.3343	0.3535	0.3629	0.3818	0.3899	0.3875
T98_R2	0.3354	0.3543	0.3634	0.3790	0.3878	0.3868
**T98_R3**	0.3710	**0.4116**	**0.4672**	0.4086	0.4511	0.4648
T101_R1	0.3590	0.3859	0.3859	0.4289	0.4289	0.4289
T101_R2	0.3239	0.3945	0.4132	0.4294	0.4408	0.4408
T101_R3	0.3258	0.4109	0.4109	0.4536	0.4536	0.4536

## Discussion

### Overall team results

As shown in Table 3, TAP-*k* scores vary significantly between team submissions, ranging from 0.3297 to 0.0296 for *k*=5. This demonstrates that the gold standard annotations of the 50 documents are capable of comparing and ranking team submissions, which indicates that the use of the proposed EM algorithm is successful in choosing a useful subset of documents (50 in this case) when it is not feasible to obtain human annotations on the entire test set for evaluation purposes. With regard to the causes for teams to produce the most divergent results on these 50 documents, we analysed and reported the number of annotated gene ids (Table 1), species distribution (Table 2) and percentage of articles with different number of species (Figure 1). We believe it is the combination of all these factors that led to the most variable results.

Overall, the results in Tables 3 and 4 suggest that this year’s GN task is more challenging compared to the past BioCreative GN tasks. The highest TAP-*k* scores on the gold standard were 0.3297 (*k*=5), 0.3538 (*k*=10), and 0.3535 (*k*=20), in two different runs submitted by the same team. To more directly compare GN 2010 team results against the ones in the past, we computed the best *break-even point*, a single value where precision becomes equal to recall, on team submissions and compared it to the best F values obtained in BioCreative I and II. The maximal break-even points are 0.41 and 0.50 on the gold and silver standard, respectively. In comparison, the best-reported F-measures on GN tasks in the BioCreative I and II range between 0.79 and 0.92, depending on the target species in question. Lower overall performance in BioCreative III can be attributed to the complexity of full text processing and species identification [15,16].

### Quality of silver standard

The development of a silver standard serves two purposes in this work. First, it enabled us to investigate how feasible it is to use a silver standard in comparing team performance compared to the use of a gold standard. As we compare team results on the same set of 50 documents using gold vs. silver standard in Table 3 and 4 (columns 2 to 4), we see that relative rankings tend to be largely preserved and the two sets of scores are highly correlated (Pearson correlation coefficient r = 0.82, 0.87, 0.89 for k=5, 10, and 20 respectively) in this comparison. For instance, teams 83, 74, 98 and 101 consistently remain as the top tier group in both evaluations. This provides some justification for the silver standard and suggests that we could use this EM algorithm approach to detect relative performance differences without any human annotations. It is noted that TAP scores are consistently higher when evaluated on the silver standard compared to the gold standard and that individual team rankings may be slightly affected. For instance, as mentioned earlier the best performing run was T83_R3 using the gold standard but T74_R3 using the silver standard.

Second, in addition to the evaluation results on the subset of 50 documents, the silver standard allowed us to assess or estimate team performance on the entire set of test articles without having human annotations for all articles. As can be seen, TAP-*k* scores in Table 4 show that overall team performance is lower on the 50 articles (columns 2 to 4) than on the entire set of 507 articles (columns 5 to 7). The reasons for this are two fold. First, the 50 articles are the more difficult ones for gene normalization and this supports our rationale for choosing them to compare team performance. As mentioned above, by comparing the gold and silver results for the 50 in Tables 3 and 4 (columns 2 to 4), we can see that team results are always higher when evaluated using the silver standard. Taken together, this suggests that the true TAP-*k* scores on the entire test set should be slightly lower than what is currently reported using the silver standard in Table 4 (columns 2 to 7), but higher than those in Table 3.

As mentioned earlier, the only way the gold standard is used in obtaining the silver standard is that we selected from each team their submission which performed best on the gold standard and then we used these team submissions to derive the silver standard. We did this with the idea that the best performing submissions on the gold standard would arguably give the best silver standard. This seems to us a necessary thing to do (why would we choose a submission that performed at a lesser level to represent a team?). But note that we did not determine nor use the team rankings on the gold standard in this step. Once we have these submissions the process of determining the silver standard was carried out without use of any information about the gold-standard human annotations. They did not enter the calculation or have any influence on how the different submissions were weighted in the EM algorithm. This is contrary to a possible concern that there is a dependency or correlation between the gold and silver standards artificially introduced by the EM algorithm. Actually, the gold and silver standards are fundamentally different because of the distinct mechanisms of how they were produced as well as how the EM algorithm was used in the process: the gold standard is human annotation of the 50 articles that were chosen by the EM algorithm with the least agreement between teams. On the contrary, the silver standard produced by the EM algorithm depends on agreements and is most influenced by the data where agreement between different teams is high.

The way we produced the silver standard by the EM algorithm is much like majority voting in that a label is more likely to be correct if returned from more *independent* sources, but it also depends also on weighting the votes by how much a particular team submission agrees with the others in the process. Thus the evaluation on the silver standard representing a team will be ranked by how much it agrees with the other submissions over the whole set of 507 test articles. Because of this, one may argue that such an evaluation approach favors methods that agree more with the norm and that it penalizes correct but deviant results. As a result, such an evaluation approach may hinder innovation rather than foster it. We would like to point out that the validity of the approach depends on the independence of the annotations produced by the different methods and not on whether one or more of the annotation methods is especially innovative. A superior method may produce annotations not produced by any other method, but if the methods are independent it will still agree more with the other methods than they do among themselves and it will likely achieve a higher rating. Independence is a common assumption that is seldom strictly satisfied, but it is often close enough to true that methods based on it are found to work well in practice. A notable example is the independence assumption that underlies the naive Bayesian classifier. It is generally agreed that this assumption is only an approximation, but it frequently works quite well [17,18]. Additionally, we refer our readers to known evidence from the machine learning literature where methods like ours have been shown successful in polling true labels for a number of similar tasks including the Amazon Turk task [6,10] and are generally robust to both noisy and adversarial labelers [6,7,9]. Indeed, our own experiments here also confirmed this conclusion, as we found that the ratings by the two methods of evaluation (gold and silver standards) are significantly correlated. How do we explain this? We are not surprised and make the claim that good systems produce correct answers more than poor systems and so they tend to agree more with other systems than poor systems and get ranked higher by the silver standard. Likewise good systems tend to get the correct answers more and so tend to agree with the human gold standard more and so get ranked higher.

Further evidence for this view comes from a direct comparison of the silver standard with gold standard annotations. Of the 507 test documents, 120 have received partial annotations by the National Library of Medicine (NLM) indexers post-challenge and these annotations mark 179 gene ids as positive and 135 of these are annotated as positive by the silver standard for a recall of 75%. Since the NLM annotations are only partial a precision figure cannot be estimated. For the 50 document gold standard only 48 documents have actual gene ids annotated. On these 48 documents the gold standard marks 1667 gene ids positive and the silver standard 1813 and the overlap is 528 for a recall of 32%, a precision of 29% and an F1 of 30%. These results suggest that the 48 document gold standard annotations are more difficult than the gold standard NLM annotations (which are generally restricted to genes central to a paper), but otherwise are consistent with the view that the silver standard is a reasonable estimate of what the gold standard annotations for a document should be. However, these results also suggest a limitation of the silver standard as it seems unlikely the silver standard is of sufficient quality to provide training data on which to base further system development and improved system performance.

### Combined performance

To test the hypothesis that combined team results can achieve better performance than the best results of a single system, as demonstrated in past BioCreative challenges [1,19,20], we built a composite system using machine learning, based on the best run submitted by each team. We experimented with various numbers of features as follows. In our first design, we represented each gene in team output by a vector of 14 binary features which correspond to its presence or absence in 14 team outputs. That is, the value of a feature *i* for gene *G* is one if *G* is predicted by Team *i*; zero otherwise. In addition to using the presence and absence of a gene prediction, we created new features to take advantage of predicted confidence scores in team output. Specifically, we add *N* features for each of the 14 binary features by stratifying each team’s predicted scores (level of confidence) into *N* groups from low to high. Two values of *N* (5 and 10) were examined separately.

Leave-one-out cross validation was conducted on the 50 articles with the gold standard annotations. That is, we train a classifier with 49 articles and test it on the remaining one article. In training, each predicted gene was labelled as a positive instance if found in gold standard annotations; negative otherwise. For testing, we used the predicted scores from our learner, an SVM like classifier with modified Huber loss function [21], to rank the genes for TAP-*k* scoring.

As shown in Table 5, the composite system achieves higher performance than the best results from any single team. Moreover, confidence scores in the predicted results are shown to be useful for further boosting performance. Comparing the numbers in the table below, we can see a 10%, 26%, and 28% increase in performance when combining team results in TAP 5, 10, and 20, respectively.

**Table 5:** TAP scores of machine learning experiments in combining team submissions in the composite system.

Systems	TAP-5	TAP-10	TAP-20
Best team result	0.3297	0.3538	0.3535
Composite system with 14 features (N=0)	0.3527	0.4241	0.4435
Composite system with 84 features (N=5)	0.3594	**0.4465**	**0.4522**
Composite system with 154 features (N=10)	**0.3614**	0.4318	0.4454

### Summary of team methods

Based on individual team descriptions below, we found that, in general, teams approached the GN task by the following steps:

1) Identifying gene mentions

2) Identifying species and linking such information to gene mentions

3) Retrieving a list of candidate gene ids for a given gene mention

4) Selecting gene ids through disambiguation and normalization

Overall, a hybrid use of rule-based and machine learning methods was observed in team descriptions. Heuristic rules were mostly developed and implemented in an ad-hoc and custom manner. With respect to machine learning tools, a number of existing packages were used including Classias [22], WEKA [23], and Mallet [24]. Further, we examined public tools and resources that were used by different teams in the four specific steps. As can be seen in Table 6, for the critical task of identifying species, Linnaeus [25] seems to be the only software publicly accessible at the time. However, as mentioned by several teams (see details in team descriptions below), simply applying Linnaeus was not sufficient to address the challenge in species recognition due to the ambiguity in species names and taxonomy ids. Furthermore, teams noted that linking gene mentions to species is difficult because a species may not be explicitly mentioned in the surrounding text. Though certain rules were helpful [16], there remains much room for future improvement. Hence, results of this GN task suggest that more community-wide effort needs to be spent on inter-species gene normalization.

**Table 6:** Public software and resources used in the GN task.

Step	Software	Other Public Resources
1)	ABNER [26] AIIAGMT [27] BANNER [28] GNAT [29] NERsuite [30] LingPipe [31]	Entrez Gene [32] Gene and Protein Synonym DataBase (GPSDB) [33] Unified Medical Language System (UMLS) [34] UniProt [35]

2)	Linnaeus [25]	NCBI Taxonomy Cell Line Knowledge Base (CLKB) [36,37]

3) & 4)	Entrez Gene [32] Lucene [38]	BioThesaurus [39] GeneRIF annotations Gene Ontology (GO) annotations & predictions [40] MeSH annotations OMIM records

It is also worth noting that as a result of this challenge, a number of new GN software and Web tools have been developed and made publicly available to the research community by participating teams. For example, three top-performing teams respectively delivered GenNorm [41], GeneTUKit [42], and IASL-IISR Gene Mention/Normalization Tool [43].

### Individual system descriptions

Each team agreed to contribute a brief summary of the most notable aspects of their system. The team summaries are given below ranked by the team’s best performance in TAP-5 on the gold standard.

#### Team 83 (Hung-Yu Kao and Chih-Hsuan Wei)

We developed an integrated method for the cross-species gene normalization on full-text articles. The proposed method consists of three modules, i.e., gene name recognition module; species assignment module; and species-specific gene normalization module. The details of the three modules are as follows.

The first is the gene mention recognition module. In this process, we use the AIIA-GMT [27] system to recognize the named entities. Due to the varied naming styles of gene names in the biomedical literature, a tagged entity cannot always exactly match a gene name in the dictionary. To address this issue, post-processing techniques that applied several translation rules on specific contexts (e.g., the number type, conjunctions, enumerations) to tokenize gene names are proposed to enhance the performance of the general-purpose gene name recognition.

The second is species assignment. The collection of species name lexicons includes three lexicons: the NCBI taxonomy, Cell line list from Wikipedia and Linnaeus corpus [25]. Synonyms of species names in the lexicon are used to detect the species name by dictionary-based matching. To handle two ambiguous cases of matching results, we devised two robust inference strategies. (1) Guaranteed inference: entities as complete names are guaranteed to indicate the Taxonomy ID and can be used to disambiguate entities, such as genus names, cell names and abbreviations, that are unguaranteed. Unguaranteed entities always occur with the guaranteed name in articles, e.g., “Arabidopsis” is accompanied by “Arabidopsis thaliana” and “HIV” by “Human immunodeficiency virus”. Conversely, “HIV” can’t imply “Human immunodeficiency virus” when this article does not contain the complete species name of “HIV”. (2) Inference by co-occurrence: the species sub-type can disambiguate the species name and genus name which appeared in the same sentence. For example, “mg1655” can disambiguate “E. coli” and “Escherichia” to the exact species (Taxonomy ID: 511145). Each previously identified gene entity is then to be assigned a suitable species ID. Several species ID assignment rules are applied for species assignment. The design of these rules was originated from Wang’s study [16]. Then, we added two rules in this module. The first rule is identifier extraction. If gene identifiers are mentioned in an article, these identifiers can be matched to their own species directly. The second rule is based on judging the lowercase letter of species. The first lowercase letter of the gene name could be an abbreviation of its species, such as a human gene “hZIP 2”.

Lastly, the proposed species-specific gene normalization module, based on our previous work [3], is utilized to calculate the inference scores for candidate Entrez IDs from articles. Two inference estimations, i.e., the entity inference and the bag-of-words inference, are used to measure the inference confidence scores. The two inference estimations both applied TF-IDF based inference networks to determine the possible Entrez IDs for each article.

#### Team 98 (Minlie Huang and Jingchen Liu)

Our system has four main modules. The first module is for gene mention recognition, the second one for gene ID candidate generation, and the third one for gene ID disambiguation. In the fourth module, the system generates confidence scores for each gene ID, where the confidence score indicates the strength of the association between a gene ID and the document.

The first module has four independent NER components: (1) a component extracting the text labeled by the <itac> tag (text emphasized in italic fonts) in html codes, with species names removed; (2) a gene mention recognition module based on a CRF that was trained on the training data from the BioCreAtIvE II Gene Mention Recognition task; (3) a dictionary-based gene mention recognition module, which was compiled from Entrez Gene; (4) the ABNER system, which is an open source NER system for biomedical text. The gene mention results from different components were merged by retaining those gene mentions that come from at least two components, but the results from <itac> tag was always kept due to its high precision. The overlap of gene mentions was chosen if their boundaries are different.

The second module generated gene ID candidates for each recognized gene mention. Lucene was used to index the gene names in Entrez Gene. Each gene mention was searched with Lucene to get the top 50 gene IDs as its ID candidates. In both indexing and searching, the gene names from Entrez Gene and the gene mentions from text were normalized using a set of rules: (1) removing all special characters such as dashes and underscores; (2) removing stop words; (3) separating at the positions where lowercase changes to uppercase, such as ‘hBCL’ into ‘h BCL’; (4) separating the digitals, Greek letters (alpha, beta etc.), Roman numbers from other letters; (5) converting to lowercase.

The third module ranked the ID candidates for each gene mention using a learning-to-rank algorithm. The training data was built from the 32 training articles with full annotation. For each gene mention recognized in the training article, the gene ID candidates were compared with the gold standard. If a gene ID is in the gold standard, it is marked as a positive example; otherwise negative. The detailed information for each gene was obtained from Entrez Gene, and features were extracted, including (1) species information (based on a dictionary lookup method), such as the frequency of this gene’s species occurring in the full text, and whether the nearest species in the context matches its gold-standard species; (2) similarity between the gene mention and the gene’s names, which was evaluated by edit distance and the score from the Lucene index engine respectively; (3) context information, such as similarity between the abbreviation or full name for the gene mention and the gene’s names, whether words indicating the gene’s function (death, binding, interacting etc) appear in the context. We tried to rank the correct gene ID to the top positions of the gene ID candidate list.

The last module output a list of final gene IDs and provided a confidence score for each gene ID. Two strategies were attempted to generating the output gene ID set. One was only keeping the top ID, the other was among the top 10 gene IDs for a gene mention, keep the top gene id for each species involved. Then we used a supervised classification method to decide the confidence scores for the gene IDs. The training data was built similarly as mentioned before: the system was run on the 32 fully annotated training articles until the output gene ID set was produced, then positive and negative examples were labeled according to the gold standard. Each gene ID has a set of features, including (1) the features used in the previous learning-to-rank module; (2) the information about the gene ID’s gene mentions, such as their minimum word number; (3) statistical information of the gene ID in the full text, such as the number of the gene ID’s gene mentions in the full text. We experimented with two models to perform the classification respectively: Logistic Regression and Support Vector Machine (SVM). We used the probability of a gene ID being positive, given by the classification model, as the gene ID’s confidence score in the final output.

#### Team 74 (Cheng-Ju Kuo and Chun-Nan Hsu)

Previously, gene normalization (GN) systems are mostly focused on disambiguation using contextual information. An effective gene mention tagger is deemed unnecessary because the subsequent steps will filter out false positives and high recall is sufficient. However, unlike similar tasks in the past BioCreative challenges, the BioCreative III GN task is particularly challenging because it is not species-specific. Required to process full-length articles, an ineffective gene mention tagger may produce a huge number of ambiguous false positives that overwhelm subsequent filtering steps while still missing many true positives.

We present our GN system which participated in the BioCreative III GN task. Our system applies a typical 2-stage approach to GN but features a soft tagging gene mention tagger that generates a set of overlapping gene mention variants with a nearly perfect recall. The overlapping gene mention variants increase the chance of precise match in the dictionary and alleviate the need of disambiguation. Our GN system achieved a precision of 0.9 (F-score 0.63) on the BioCreative III GN test corpus with the silver annotation of 507 articles. Its TAP-k scores are competitive to the best results among all participants. We show that despite the lack of clever disambiguation in our gene normalization system, effective soft tagging of gene mention variants can indeed contribute to performance in cross-species and full-text gene normalization.

#### Team 101 (Richard Tzong-Han Tsai and Hong-Jie Dai)

In our system, we employed a method modified from [44]. It includes a multi-stage GN procedure and a ranking method which exploit information from different parts of a paper. Unlike abstracts, full text articles contain several sections. Each section of the paper has different characteristics which we can use to guide GN and the ranking algorithm. For example, the introduction is usually the richest section because it is here that authors first mention the genes of interest, give their full names, and often their abbreviations to be used thereafter. The least informative sections tend to be figure/table captions, which lack context information. Our multi-stage GN procedure is carried out starting from the sections with the richest context information (introduction) to those with the poorest.

Since the species in a context is unknown, all entries in the gene name dictionary must be loaded for GN. Entrez Gene, the largest and most widely used publicly available gene or gene product database, has the best coverage of names and species. However, it contains millions of names, which if all loaded for GN, may greatly slow down the GN process. We reduce ambiguity by selecting dictionary entries only from the top 22 most common species in NCBI (from 7283 species). Comparing the scores of our system to the average scores of the BioCreative III GN participants, our TAP-*k* score exceeds the BioCreative III average from 24% to 70%. According to the official results, our submission consistently remains in the top tier group in all evaluations [45].

#### Team 93 (Naoaki Okazaki and Han-Cheol Cho)

Our system recognizes gene mentions in source articles using NERsuite [30] trained with the corpus of the BioCreative II Gene Mention Recognition task and gazetteers from UMLS [34] and Entrez Gene. For each recognized gene mention, the system enumerates candidate gene identifiers (Gene-IDs), and computes the confidence score of each Gene-ID. Gene-IDs found in the article are ranked by the sum of the mention-level scores. The system also uses LINNAEUS [25] for species mention recognition.

Gene normalization assigns an Entrez Gene-ID for a gene mention. This is performed by two subtasks: *candidate retrieval* and *candidate scoring*. In candidate retrieval, we built inverted indices that associate the contents of name fields of Entrez Gene records (e.g., gene locus, gene synonyms, protein name, nomenclature full-form) with Gene-IDs. When designing this component, we prioritize recall over precision because the subsequent components cannot recover from misses (false negatives) of candidate retrieval. At the same time, it might be difficult for candidate scoring to choose a true (positive) Gene-ID from a large number of irrelevant (negative) Gene-IDs. Therefore, we introduced some heuristics to reduce the number of candidate Gene-IDs. For example, the species of the Gene record is required to be mentioned somewhere in the source article.

We score each candidate Gene-ID by using binary logistic regression. In order to train the model, we manually annotated the gold-standard mention(s) for each Gene-ID in the training sets 1 and 2. In Gene-IDs enumerated by the candidate retrieval for each gold-standard mention, the Gene-ID in the training sets presents a positive instance, and the rest present negative instances. We used Classias [22] as a tool-kit for training the logistic regression model. Features of the model are categorized into four types: *mention-name features*, *context features*, *PMID features*, and *organism features*. A mention-name feature captures orthographic similarity between a gene mention and the name fields of the Entrez Gene record. We prepared a mention-name feature for every combination of fields (e.g., gene synonyms and protein names) and matching methods (e.g., exact match, approximate match, letter n-gram similarity). Context features compute the cosine similarity between the surrounding expressions (context) of a gene mention and descriptions (e.g., summaries and GeneRIF texts) in the Entrez Gene record. We designed a context feature for each window of context (abstract, title, titles of references, paragraph, sentence, preceding five words, and succeeding five words) and for each descriptive field. A PMID feature indicates whether the Entrez Gene record includes the PMID(s) related to the target paper (the PMID of the paper and PMIDs of the related work) in the reference of the record (e.g., PMIDs appearing in GeneRIF information). An organism feature examines whether the species of the Gene record appears in a context window of the mention. Here, we use the results of the species mention recognizer to link taxonomy identifiers (TaxIDs) and context expressions (e.g., TaxID 9606 and the expression *patients*).

#### Team 68 (Martin Gerner and Illes Solt)

##### Summary

We present an ensemble system encompassing LINNAEUS for recognizing organism names and GNAT for recognizing and normalizing gene mentions. Candidate gene identifiers are filtered and scored through a series of steps that consider the mention itself, its local context, as well as general knowledge about each candidate gene.

##### Methods

To determine the set of species related to a document, we analyze its associated MeSH terms for occurrences of species names using the LINNAEUS system [25]. LINNAEUS uses a dictionary of expanded species terms from the NCBI Taxonomy, together with rule-based methods and distributional statistics to disambiguate ambiguous species mentions. To further increase the utility of LINNAEUS for detecting organisms related to articles, even if they are not mentioned directly, we have included additional "proxy" dictionaries that associate cell-lines and genera to species. The cell-line dictionary was created from the CLKB [37]. Genera mentions are linked to the member species that is most commonly mentioned in MEDLINE (for example, *Drosophila* is linked to *Drosophila melanogaster*).

Gene name recognition and mention normalization are handled by GNAT [29]. To recognize gene mentions, GNAT performs species-dependent NER; that is, for every species found to be related to a text, it runs an independent dictionary-based NER module, one by one. Dictionary entries consist of gene and protein names from Entrez Gene and UniProt, with the corresponding identifiers, where each synonym was expanded into a regular expression that covers orthographic, lexical, and structural variations. The dictionary entries provide candidate Entrez Gene IDs for gene mentions, which we consider in the subsequent disambiguation steps.

We perform a series of filtering steps to reduce ambiguity and remove likely false positives. These filters take into consideration the gene mention itself, for instance, excluding names that more likely refer to a disease; a gene mention's immediate context, that is, words to the left and right that might be indicative of a non-gene name; and the overall context of the gene mention, usually the paragraph the name occurs in, which we compare to general knowledge about each candidate gene, such as Gene Ontology terms, sequence features, known implications in diseases, and so on. As the last step in our filtering pipeline, we map genes to species; we use heuristics that locate the closest species mention, looking in the same sentence, paragraph, article title, abstract, and finally the full text as well as MeSH terms if available.

Gene identifiers are scored based on a number of factors, the main ones being: *i*) whether it is ambiguous, *ii*) its string similarity to the corresponding gene symbol, and *iii*) the number of times the same text fragment appeared in italics throughout PubMed Central. To compile a ranked gene list for TAP computation on the document level, mention-level candidate identifier scores are aggregated.

##### Results

On the BioCreative III GN high-quality training data, our system achieves TAP-5 and TAP-20 scores of 0.36 and 0.41, respectively. The performance on test set dropped significantly to 0.16 and 0.20. Our system's thresholded predictions achieved precision, recall and F1-scores of 0.54, 0.47, and 0.50, respectively on the training data, and 0.33, 0.15 and 0.21 on the test data.

##### Conclusions

For BioCreative III, we optimized gene NER precision by limiting the set of species (gene name dictionaries) to the most frequent ones. These 22 species cover about 69% of all species mentions in MEDLINE and PubMed Central, and accounted for 95% of all gene mentions in the training data, but for only 44% on the test data. Our analysis of the evaluation results suggests that the scores primarily drop due to this significant difference in species composition; and partly due to the way in which evaluation data were selected, affecting the results of all participants equally.

#### Team 89 (Shashank Agarwal and Feifan Liu)

We developed a three-tiered GN system, as described below. In the first tier, the goal was to identify gene mentions in an article with maximum recall. Gene mentions were identified using two methods. In the first method, an existing Conditional Random Fields (CRF) based gene named entity recognizer, BANNER [28], was adapted to identify gene mentions. The second method to identify gene mentions was based on italics markup tags in the document. The data provided for BioCreative III was in XML format, where italics markup is usually available for gene/protein names and species name. All terms marked in italics were added as gene mentions to maximize recall despite leading to more false positives at this point.

In the second tier, the goal was to identify candidate gene IDs for identified gene mentions. We built an index of all gene symbols and names in the Entrez Gene database and linked them to the corresponding gene ids using Apache Lucene. Each gene mention identified in Tier 1 was first expanded by a rule-based gene name variation generator and then used to query the Entrez Gene index. Top 100 IDs returned as a result to each query were considered as the candidate IDs for the corresponding gene mention.

In the third tier, the candidate genes were disambiguated in a learning framework, and several learning algorithms in the Weka toolkit [23] were explored and evaluated. Twenty-seven features were identified for each candidate gene, including presence of gene’s species in the article (species in article were identified by LINNAEUS [25]), presence of a part or whole of the gene’s genetic sequence in the article, edit distance and Jaro-Winkler similarity between the gene’s synonyms and the identified mention, and similarity between the gene’s GO and GeneRIF annotations and the article. The disambiguation task was formulated as a binary classification task, aiming to determine whether each candidate gene is a correct mapping or not based on features defined above. Random Committee and Random Forest were found to attain the best performance. We also investigated using Chi-Square based feature selection, adding training instances from important gene annotations, expanding output with negative prediction based on confidence scores as well as system combinations.

#### Team 80 (Dina Vishnyakova and Patrick Ruch)

In the BioCreative III Gene Normalisation (GN) challenge, we can split the task into three subtasks: gene name recognition, species name recognition and gene normalization. In the first subtask we face ambiguity of gene names, dealing with homonyms and synonyms. In the second subtask we deal with species name detection, considering that species are often implicit. The third subtask consists of using gene names to assign species.

For the GN task we generated three runs. All three runs shared similar methods of gene name recognition (GNR) and species detection but different methods of gene normalization. We use GPSDB [33] as a dictionary to filter gene candidates detected by our Gene Name Recognizer and to assign an identifier to every identified gene name. In our post-competition analysis, it appeared obvious that our results are dependent on the confidence score provided by the system. The confidence score in our system is based on species and gene name weighting.

In order to detect species’ names we used simple rule-based approaches and created recognition modules for a dozen of the most common model organisms, such as human, mouse, fly, etc. The choice of these species was based on observations made on the training set as well as on the distribution found in GPSDB.

In run 2 and run 3 the score of every gene identifier in the result list is highly dependent on the species name scoring. Run 2 weights species based on species occurrences in the title of articles, sections and subsections. Run 3 considers species distributions across the whole article. As for the results of run 1, they are not only dependent on the species weighting but also on the results of the Gene Ontology categorizer (GOCat) [40,46]. What makes this run comparatively more effective is the use of GOCat’s functional prediction power to assign protein identifiers. We compute a similarity measure (lexical and nearest neighbour distances) between articles and functional descriptors in Entrez Gene. Using results provided by GOCat we filter gene candidates and give additional weight to those candidates. GOCat not only helps to filter gene candidates but also often to simply boost positive gene identifiers already assigned by the gene name entity recognizer.

All combinations between the modules were set empirically on training data. The impact of GOCat appeared more effective on the official data than on our training runs. This impact suggests that overfitting phenomena are avoided mainly because of GOCat which was not originally designed for the gene recognition. After competition, for run 3 we have improved our results for the TAP-5 by +312%, TAP-10 by +219% and TAP-20 by +152.7% on the “gold50” standard. In addition to this we have improved results by +24.5% for the TAP-5, 10, 20 of the “silver507” standard. For run 1 we have improved results for the TAP-5 by +12.3%, TAP-10 by +10.4% and TAP-20 by +11.5% of the “gold50”. For the TAP-5, 10, 20 of the “silver507” we have obtained an improvement of +5.3%. We noticed that our system was able to find gene identifiers for only 443 out of 507 documents. This can be explained by our incomplete vocabulary of organisms, e.g. viruses. Our GN system is available online at http://pingu.unige.ch:8080/NormaGene.

#### Team 65 (Martin Romacker and Fabio Rinaldi)

The OntoGene research group at the University of Zurich used for the GN task a variant of their text mining pipeline which had been previously developed for the detection of protein-protein interactions [47,48]. While the full OntoGene system includes modules for syntactic parsing and relation extraction, for the GN task a simplified version of the pipeline was used, including modules for conversion into the internal XML format, preprocessing (sentence splitting, tokenization, tagging), terminology recognition, detection of ‘focus organism’, terminology filtering and scoring.

The terminology recognition module is based on an efficient lexical lookup approach, with the contribution of a ‘normalization’ module (rule based) which can take into account the most frequent surface variants of a term. The lookup uses an internal terminological resource built using terms extracted from UniProt, Entrez Gene, NCBI Taxonomy, Cell Line Knowledge Base (CLKB) [36]. Entity resolution is based on a terminology filtering and scoring approach, which is based on the one hand on textual features, on the other hand on the detected organism. It functions as follows: for each term for which a focus organism above a probability threshold filter has been identified, and which is not in a stop word list, a score based on frequency of the term, the zone (title, abstract, main text), and organism-related keywords, is calculated as follows: SCORE = f * org, where

• f: frequency of term in text (an occurrence in the title has a weight of 200, an occurrence in the abstract a weight of 8; additionally terms in italics are weighted 3 times higher).

• org: organism score from “focus organism” detection module (rebalanced through some specific additional organism-related keywords).

The difference between our two submitted runs is mainly in the terminological resources. RUN 1 did not use Entrez Gene or UniProt, but instead used an extensive terminological resource provided by TMS (Text Mining Services, Novartis AG), which, however, covers only the five most important model organisms (human, mouse, rat, yeast and drosophila). Additionally, we included organism resources extracted from the NCBI taxonomy and terms from the CLKB. The TMS resource contains 670,000 term senses. Our own organism and CLKB resource contains 49,000 term senses. This resulted in 520,000 normalized terms, and 172,000 different gene IDs from 5 different organisms.

RUN 2 additionally used 2,203,000 terms from UniProt (version from June 2010) and 1,021,000 terms from Entrez Gene (only 20 topmost organisms from the training data, for efficiency reasons). This resulted in 1,856,000 normalized terms and 833,000 different gene IDs from 2,113 different organisms.

The official results show that the resource used for RUN 1 appears to be sufficiently complete, in comparison with the subset of Entrez Gene used for RUN 2, where we have an increase of nearly 20% FP, which can hardly be compensated by the increase of about 3.5% in TP. Unexpectedly, the TAP-*k* measures are definitely better for RUN 2. This would suggest that RUN 2 produced a better ranking than RUN 1. A possible explanation for this difference is that the contribution of the “focus organism” detection module [15] is better in RUN 2 than in RUN 1 (therefore genes belonging to the selected organisms are ranked higher). This module, which was initially developed for PPI detection, uses all relevant terminology in the article in order to derive an organism ranking: in particular terms from NCBI and CLKB, but also protein mentions. Crucially however, it does not use gene mentions to the same extent as protein mentions. Therefore the lack of sufficient protein mentions in RUN 1 produced a lower quality ranking of organism, which in turn resulted in a worse ranking for genes.

#### Team 78 (Sanmitra Bhattacharya and Padmini Srinivasan)

Our approach described in [49] is similar in some aspect to others that employ gene and species identification and their association for cross-species gene normalization. For our GN system we experimented with two widely used gene name taggers namely ABNER [26] (trained on the NLPBA corpus) and LingPipe [31] (trained on the GENIA corpus). The gene mentions identified by these systems were filtered using a stop list of terms like antibody, *Ab*, antigen, *IgG*, etc. Shorthand gene names were expanded to constituent terms as in *Xnr1*, *Xnr2…Xnr6* for *Xnr1-6*. LINNAEUS [25] was used for species name identification. However, the species dictionary had to be modified for inclusion of *genera* of model organisms and commonly occurring species strains (e.g. *Saccharomyces cerevisiae S288c*). The gene and species names identified were associated based on proximity within fixed character windows. Different combinations of the above-mentioned taggers and different association strategies were used to set up various experiments. We selected three strategies for the test submission based on their performance on the training data.

For the first strategy, a gene name (identified using LingPipe only) is associated with a species if it is found within a specified character window. If an association is not found in the given window then the species occurring most frequently in that article is associated with the gene name. Confidence was calculated either from the proximity of the gene and species mentions or from the percentage of the majority species. For the second strategy, in an effort to increase the precision of this strategy, we used both ABNER and LingPipe while keeping the other settings identical. The gene names identified using ABNER and LingPipe were associated with the species names identified by LINNAEUS within a fixed character window. From these two associations, only the overlapping ones were considered along with the higher confidence score. In the third strategy, we considered only the intersection of gene-species associations (gene names identified by ABNER and LingPipe) within a fixed window of much larger size compared to the previous strategies.

For each of the gene species associations, we search Entrez Gene [32] for a unique identifier for that gene mention. The first Entrez Gene identifier retrieved from this search was returned as the unique identifier for that gene name. The highest scores for the “gold50” standard were achieved using the second strategy where the TAP-*k* (k=5, 10, 20) scores were 0.0847, 0.1202 and 0.1706, respectively. However, post-submission analysis revealed that limiting the Entrez Gene search to some specific fields like official gene/protein name, official symbol and synonyms would improve the results. Incorporating these changes, our best scoring strategy now achieved TAP-*k* scores of 0.1915, 0.1928 and 0.1930, respectively. Another strategy similar to the second strategy, but one considers union instead of intersection of ABNER and LingPipe identified genes, gave us even higher TAP-*k* (*k*=5, 10, 20) scores of 0.1848, 0.2172 and 0.2172, respectively. These improvements are in the range of 118.18% (TAP-5) to 27.31% (TAP-20) compared to our best submitted runs.

#### Team 97 (Hongfang Liu and Manabu Torii)

The team participated in gene normalization tasks in all three BioCreative workshops and used the same approach to tackle the gene normalization task: a comprehensive list of synonyms and a flexible dictionary lookup method. In BioCreative I, the team achieved the best recalls among the participating systems for yeast and mouse but the precisions were very low. For BioCreative II, the team applied a machine learning module following the dictionary lookup to filter out false positives and this system achieved the first quartile performance. In BioCreative III, the machine learning module used in BioCreative II was not applied due to the high ambiguity associated with all species gene normalization. Therefore, heuristic rules based on features obtained for each pair (Term, GeneID) were used, where Term is a phrase in text and GeneID is a potential gene identifier for the Term. Specifically, for each pair (Term, GeneID), we derived a descriptive feature vector to represent i) ambiguity and systematic ambiguity features of Terms based on BioThesaurus and GeneRIF, ii) document-level taxonomy assignment counts based on a machine learning taxonomy classifier trained using GeneRIF, iii) counts of GeneID in the document, iv) number of synonyms representing GeneID, and v) whether Term is detected by gene mention systems or not.

#### Team 70 (Sergio Matos and David Campos)

The GN system used for this challenge is composed of five modules: corpus pre-processing, named entity recognition (NER), dictionary-lookup, context-matching, and rule-based decision [50]. For NER, we trained a Conditional Random Fields (CRFs) model using Mallet [24] with the BioCreative II Gene Mention corpus as the training data set. The set of features includes: word stemming, part-of-speech tagging, orthographic and morphological features, Greek letters, dictionary-matching of gene/protein names, relevant verbs and other biological concepts such as nucleobases, amino acids and nucleic acids. Local context was modeled with a {-1, 1} window of features.

Each mention identified by the NER module is mapped to possible Entrez Gene identifiers through dictionary-lookup. To accomplish this task and to achieve efficient approximate string search, we used Lucene [38] to index a lexicon of gene/protein names, using the BioThesaurus [39] resource as the starting lexicon. This dictionary was extended with some lexical variations of each gene name using various string-editing rules: removing dashes; replacing dashes by spaces; inserting a dash on a letter-digit sequence; replacing Arabic numerals by roman numerals (and vice-versa); replacing Greek letter names by their initial (e.g. *alpha* → *a*).

The following step is to calculate a measure of likelihood for each of the candidate gene IDs. This is achieved by comparing the local context of each mention found in the text to knowledge profiles associated to each gene ID. These profiles were created by merging the descriptive fields extracted from the UniProt, Entrez Gene and OMIM databases, as well as the Gene Ontology terms associated with each gene entry, and implemented as a free-text index (separate from the dictionary index). Therefore, to obtain the confidence scores for a gene mention, we search this index using the sentence where the mention occurs. The resulting identifiers are then cross-matched with the candidate gene IDs obtained in the dictionary-lookup step. This step is performed at the document level in order to accumulate the confidence scores across different mentions of the same gene. This can either be a repetition of the same gene name in the document, or the occurrence of a synonym for the same gene (for example, occurrences of “pten induced putative kinase 1” and “pink1”).

Finally, the last step performs the disambiguation between the candidate gene IDs in a document. All possible identifiers obtained after context matching are assembled, and the most likely identifiers are selected according to some empirically created rules. The rules select identifiers matched with more than five mentions in text, or matched to at least three mentions and with an average context-matching score equal to or higher than a threshold (set at 0.8). Other identifiers matched to the same text mentions but having lower scores are rejected. This step also rejects gene mentions with low confidence scores, in order to eliminate some false positives obtained during the entity recognition step.

#### Team 63 (Karin Verspoor and Kevin M. Livingston)

The University of Colorado School of Medicine submission to the BioCreative III gene normalization task experiments with a novel knowledge-based approach to the problem. The approach, called *Knowledge-based Normalization of Gene Mentions* (KNoGM) follows the paradigm of knowledge-based solutions to word sense disambiguation (WSD) in general English [51]. To cast the gene normalization problem as a WSD problem, we treat gene mentions as the ambiguous words, and the Entrez Gene identifiers corresponding to each gene name as the word senses.

The method employs a graph search algorithm known as *personalized PageRank*[52], based on the well-known link analysis algorithm *PageRank*[53]. The intuition underlying the approach is that the structure of the relationships among concepts drives the relative importance of a concept. Further, by taking co-occurrence in a document of related concepts into consideration, the most salient concepts within the document context can be identified. As an example, consider an ambiguous word such as “cell”, which in general might refer to either a terrorist unit or a structure containing biomolecules. The terrorist sense would be related to concepts such as *bombing*, *kidnapping*, etc. while the biological sense would be related to concepts such as *protein*, *DNA*. By taking advantage of those relationships, combined with recognition of those related concepts in the same document context as the word “cell”, we can resolve the ambiguity in context. The relationships among concepts defined in a knowledge graph drive the analysis, rather than statistics computed over words in a training corpus.

To build the knowledge graph, we take advantage of the large number of curated sources of formal biological knowledge that are available. Nodes in the graph are biological concepts, and links are associations among those concepts. Specifically, we include associations between genes and their gene products (proteins) and direct protein interaction information, in addition to associations to concepts which indirectly relate genes to each other, such as Gene Ontology [54] annotations, and organism information through NCBI Taxonomy terms. For our solution, we built a knowledge graph from public data sources, including Entrez Gene, UniProt, iRefWeb [55], GOA [35], and HomoloGene [56]. This knowledge graph is extracted from an RDF-based triple store we populated to unify the separate knowledge sources.

This approach prefers to resolve an ambiguous gene name to an identifier already known to be connected to other genes mentioned in the document based on curated biological relations.

Our system for BCIII:

(1) used the AIIA-GMT gene mention tagger [27] to identify gene mentions,

(2) mapped each mention to candidate database identifiers using the BioThesaurus v.6.0 [57],

(3) applied abbreviation detection using the Schwartz and Hearst algorithm [58] to filter the candidate sets,

(4) created document term co-occurrence windows of varying sizes, and

(5) ran the UKB disambiguation implementation (http://ixa2.si.ehu.es/ukb/) [51] utilizing the graph extracted from the triple store.

The highest-ranked sense (identifier) produced by UKB based on context was returned as the gene mention normalization. This evaluation used uniform weighting of the graph and we did not explore weighting variations.

## Conclusions

We have successfully organized a community-wide challenge event for the gene normalization task. There were a total of 37 submissions by 14 different teams from Asia, Europe, and North America. The highest TAP-*k* scores obtained on the gold-standard annotations of the 50 test articles are 0.3297 (k=5), 0.3538 (k=10), and 0.3535 (k=20), respectively. In addition, TAP-*k* scores of 0.4916 (k=5,10,20) are observed when using the silver standard of the 507 test articles.

In comparison with past BioCreative GN tasks, this year’s task bears more resemblance to real-world tasks in which curators are given full text without knowing species information. As a consequence, this year’s task has proved more difficult than the ones in the past, which is evident from the overall lower team performance.

Finally, we believe the TAP-*k* metric and EM algorithm proved adequate for evaluating retrieval efficacy and for choosing a small number of articles such that we can afford to obtain a sufficient gold standard for evaluation purposes while keeping the manual curation effort feasible. Moreover, the proposed pooling method allowed us to infer ground truth based on solely team submissions. By comparing team rankings on gold vs. silver standards, we show that we can effectively detect good team performance without having to rely on human annotations. Future work should investigate how systems developed for the GN task may be used in real-world applications and further promote community-wide efforts toward improved inter-species gene normalization.

## List of abbreviations used

GN: Gene Normalization; EM: Expectation Maximization; TAP: Threshold Average Precision; MOD: Model Organism Database; BMC: BioMed Central; PLoS: Public Library of Science; PMC: PubMed Central; NCBI: National Center for Biotechnology Information; NLM: National Library of Medicine; UAG: User Advisory Group; AP: Average Precision; SVM: Support Vector Machine; CRF: Conditional Random Fields; GNR: Gene Name Recognition; GOCat: Gene Ontology Categorizer; CLKB: Cell Line Knowledge Base; NER: Named Entity Recognition; KNoGM: Knowledge-based Normalization of Gene Mentions; WSD: Word Sense Disambiguation

## Competing interests

The authors declare that they have no competing interests.

## Authors’ contributions

ZL and WJW organized the task and drafted the paper. The remaining authors were participants in the BioCreative III Gene Normalization task and provided individual team system summaries. All authors read and approved the manuscript.

## Supplementary Material

Additional file 1 GN annotation guidelines (AnnotationGuidelines.pdf) Click here for file

Additional file 2 **Introduction to TAP-*k* (What is TAP.pdf)** The evaluation data and TAP-*k* software are freely available at the BioCreative III Website: http://www.biocreative.org. Click here for file
