# Supplementary material for: The gene normalization task in BioCreative III
Source: BMC Bioinformatics. 2011 Oct 3;12(Suppl 8):S2. doi: 10.1186/1471-2105-12-S8-S2 (PMC3269937; doi:10.1186/1471-2105-12-S8-S2)
Supplement: Additional file 1 — GN annotation guidelines (AnnotationGuidelines.pdf) [file 1471-2105-12-S8-S2-S1.pdf]

BioCreative GN Task 2010

Gene/Protein Annotation Guidelines

What to annotate and normalize:

1. Find gene/protein mentions in the full-length article including figure and table legends and map them to unique Entrez Gene identifiers (http://www.ncbi.nlm.nih.gov/gene/).
2. Entrez Gene Ids are required. (UniProt Ids or Model Organism Database Ids are optional).
3. Annotate all genes mentioned in the article including those genes mentioned in passing or only mentioned once in the article. However, there is no need to rank or group genes for this assignment.
4. When there is no explicit mention of a gene's organism of origin in surrounding text, try to use the article context to help determine its species. Annotate the gene only when the species information can be determined. Some helpful clues for determining species include details in the methods/materials section such as cell lines, organism-specific gene nomenclature conventions, etc.
5. You may also use your domain knowledge for determining which organism a gene belongs to when no explicit species information is given in the text. If there is absolutely no clue about the species, or in situations where the species information is ambiguous (e.g. the authors use one gene as a representative of its homologs), do not annotate the gene.
6. When cell lines from different species are used to study a gene, determine and use the gene's *species of origin* instead of a cell lines' *species of origin* for annotation.

What NOT to annotate:

1. Do not annotate references sections. But this section may be useful for species identification. However, do not go beyond reading reference titles. That is, don't read the referenced articles.
2. Do not use or annotate supplementary material or supporting information.
3. Annotate target proteins but do not annotate antibodies/reagents that are used to study target proteins.
4. Do not annotate the Methods/Materials section for genes/proteins. But this section may be useful for species identification. (Our reasoning is that the Methods/Materials section often contains information about reagents or antibodies that are themselves proteins but are not *curatable* objects; if *curatable* genes/proteins are mentioned in such a section, then they will almost certainly be mentioned elsewhere in the article).
5. Do not annotate genes where no unique ids can be identified in Entrez Gene. For example, if you find a gene mention "x-tsk" in a paper and subsequently search it in Entrez Gene, you may be presented with two separate Entrez gene records (x-tsk-b1 & x-tsk-b2). In this case, if you can't tell which specific gene is used in the paper based on your domain knowledge, do not annotate this gene.
6. Do not annotate a protein complex (e.g. TFTC complex). But if its members are explicitly given (NFKB-IKB complex) they should be annotated.
7. Do not annotate a protein family (e.g. cytokines; ring-h2 finger proteins) because no unique Entrez Gene id can be assigned to it.
8. Do not annotate a gene/protein with only non-species taxonomic information (e.g. mammalian p53) for the same reason above.
