# Supplementary material for: The gene normalization task in BioCreative III
Source: BMC Bioinformatics. 2011 Oct 3;12(Suppl 8):S2. doi: 10.1186/1471-2105-12-S8-S2 (PMC3269937; doi:10.1186/1471-2105-12-S8-S2)
Supplement: Additional file 2 — Introduction to TAP-k (What is TAP.pdf) The evaluation data and TAP-k software are freely available at the BioCreative III Website: http://www.biocreative.org. [file 1471-2105-12-S8-S2-S2.pdf]

What is *TAP-k*?

Here we refer to the measure defined by Carroll, H. D., Kann, M. G., Sheetlin, S. L., and Spouge, J. L., Threshold Average Precision (TAP-k): A Measure of Retrieval Designed for Bioinformatics, *Bioinformatics Advanced Access published on May 26, 2010*.

The Threshold Average Precision (*TAP-k*) is *MAP* with a variable cutoff and terminal cutoff penalty.

For a single query the average precision (*AP*) is computed by summing the precision at each rank that contains a true positive item and then dividing this sum by the number of positives for that query. If the retrieval system assigns to each retrieved item a score and the retrieved items are ranked in decreasing order of score, then it may be useful to cut off the retrieval at some fixed score level x. We can compute the average precision with cutoff x (APC_x). This is the sum of the precision at each rank with a true positive item and a score >=x, divided by the total number of positives for the query. Finally, suppose that $y>x$ and further suppose there are no true positive items in the sum for APC_x that are below y. Then $APC_y=APC_x$. But clearly it would make more sense to choose the cutoff y than the cutoff x. To distinguish between these two cases we define the average precision with cutoff x and terminal penalty ($APCP_x$). Let P_x be the precision at the last rank with score >= x and let P be the total number of positives. Then define

$$APCP_x = \frac{TP * APC_x + 1 * P_x}{TP + 1}. \tag{1.1}$$

$APCP_x$ is just the weighted average of APC_x and P_x with most of the weight applied to APC_x, but P_x supplying the terminal penalty. In our hypothetical case P_y will be greater than P_x so that $APCP_y$ is also greater than $APCP_x$ and the score rewards the better choice of cutoff or equally penalizes the poorer choice. Whereas *MAP* is the average of *AP* over all the queries, *TAP-k* is the average of $APCP_x$ over all the queries where x is chosen as the largest score that produces a median of k false positive retrievals over all the queries. The median is used here instead of the mean because it is more robust against noise and outliers.

There are some practical considerations when applying *TAP-k*. First, retrieval systems must produce scores commensurate with their rankings and these scores must be interpretable across different queries. Since most systems generate their retrieval by scoring this should not make the task any more difficult than usual. On the other hand some kind of score normalization may be necessary for some systems, depending on how the scores are constructed. An ideal score would be a probability estimate that the retrieved item is a true positive, but a score need not be a probability estimate for good performance. The score that is reported simply has to have the same implications for relevance of the item regardless of the query, for the best performance. Another important issue is the length of the retrieved lists returned by a system. If many of the retrieved lists are too short to have k false positives appear, then no cutoff score may produce a

median number of *k* false positive retrievals for the set of queries. In that case we will take the cutoff score *x* to be the lowest score over all the retrieval lists for all the queries.

Example 1. Data for five queries, Q1-Q5 are presented in the table. The numbers in parentheses following the query numbers are the number of correct or relevant items for each query. This data was generated randomly based on the scores. Each score is the probability that the corresponding retrieved item would be relevant (relevance is shown by a 1 in the rel column for each query). The scores themselves are parts of power series which are convenient for generating realistic scores. Retrieval is cut off at 15 items for each query to keep the data easily manageable and as a consequence not all relevant items are necessarily retrieved.

	Q1 (5)		Q2 (5)		Q3 (5)		Q4 (3)		Q5 (5)	
	rel	score	rel	score	rel	score	rel	score	rel	score
1	1	0.900	0	0.500	0	0.500	0	0.2	1	0.980
2	1	0.738	0	0.475	1	0.475	0	0.187	0	0.788
3	0	0.605	1	0.451	0	0.451	0	0.174	0	0.633
4	1	0.496	0	0.429	0	0.429	0	0.163	1	0.509
5	1	0.407	1	0.407	0	0.407	0	0.152	1	0.409
6	0	0.334	0	0.387	0	0.387	0	0.142	0	0.329
7	0	0.274	0	0.367	0	0.367	0	0.132	0	0.265
8	0	0.224	0	0.349	1	0.349	0	0.123	0	0.213
9	1	0.184	0	0.332	0	0.332	0	0.115	0	0.171
10	0	0.151	1	0.315	1	0.315	0	0.107	1	0.138
11	0	0.124	0	0.299	0	0.299	0	0.100	0	0.111
12	0	0.101	0	0.284	0	0.284	0	0.094	0	0.089
13	0	0.083	0	0.270	0	0.270	0	0.087	0	0.071
14	0	0.068	0	0.257	0	0.257	0	0.082	0	0.057
15	0	0.056	0	0.244	1	0.244	0	0.076	0	0.046

Here the score cutoff for *TAP*-5 is 0.213 and the values of $APCP_5$ are 0.675, 0.206, 0.264, 0, 0.413 and the average of these numbers, *TAP*-5, is 0.312. The blue background shows what parts of the retrieval were included in the scoring (likewise for subsequent examples).

Example 2. Example 1 output, but the system has limited its retrieval to the top 4 ranks for each query.

	Q1 (5)		Q2 (5)		Q3 (5)		Q4 (3)		Q5 (5)	
	rel	score	rel	score	rel	score	rel	score	rel	score
1	1	0.900	0	0.500	0	0.500	0	0.2	1	0.980
2	1	0.738	0	0.475	1	0.475	0	0.187	0	0.788
3	0	0.605	1	0.451	0	0.451	0	0.174	0	0.633
4	1	0.496	0	0.429	0	0.429	0	0.163	1	0.509
5										
6										
7										
8										
9										
10										

11										
12										
13										
14										
15										

Here the cutoff score is 0.163 (the lowest score possible) and the $APCP_5$ values are 0.583, 0.097, 0.125, 0, 0.333 and the average, *TAP*-5, of these numbers is 0.228. Here the *TAP*-5 is lower than for example 1 because the system cut the retrieval off prematurely and this decreased the recall and thus the *TAP* -5 score.

Example 3. Example 1 output again, but scores changed so they only reflect the rank and not the quality of the retrieved material.

	Q1 (5)		Q2 (5)		Q3 (5)		Q4 (3)		Q5 (5)	
	rel	score	rel	score	rel	score	rel	score	rel	score
1	1	0.9	0	0.9	0	0.9	0	0.9	1	0.9
2	1	0.85	0	0.85	1	0.85	0	0.85	0	0.85
3	0	0.8	1	0.8	0	0.8	0	0.8	0	0.8
4	1	0.75	0	0.75	0	0.75	0	0.75	1	0.75
5	1	0.7	1	0.7	0	0.7	0	0.7	1	0.7
6	0	0.65	0	0.65	0	0.65	0	0.65	0	0.65
7	0	0.6	0	0.6	0	0.6	0	0.6	0	0.6
8	0	0.55	0	0.55	1	0.55	0	0.55	0	0.55
9	1	0.5	0	0.5	0	0.5	0	0.5	0	0.5
10	0	0.45	1	0.45	1	0.45	0	0.45	1	0.45
11	0	0.4	0	0.4	0	0.4	0	0.4	0	0.4
12	0	0.35	0	0.35	0	0.35	0	0.35	0	0.35
13	0	0.3	0	0.3	0	0.3	0	0.3	0	0.3
14	0	0.25	0	0.25	0	0.25	0	0.25	0	0.25
15	0	0.2	0	0.2	1	0.2	0	0.2	0	0.2

Here the scores no longer reflect quality and thus they do not give an accurate idea of where to cut off retrieval to obtain maximal efficiency. As a result there is a drop in *TAP*-5 as compared with example 1. The cutoff score is 0.6 and the $APCP_5$ values are 0.687, 0.170, 0.107, 0, 0.421 and the average, *TAP*-5, is 0.277.
